# Survivability of *Kluyveromyces marxianus* Isolated From Korean Kefir in a Simulated Gastrointestinal Environment

**DOI:** 10.3389/fmicb.2022.842097

**Published:** 2022-02-24

**Authors:** Hye-Young Youn, Dong-Hyeon Kim, Hyeon-Jin Kim, Dongryeoul Bae, Kwang-Young Song, Hyunsook Kim, Kun-Ho Seo

**Affiliations:** ^1^Center for One Health, College of Veterinary Medicine, Konkuk University, Seoul, South Korea; ^2^Department of Food & Nutrition, College of Human Ecology, Hanyang University, Seoul, South Korea

**Keywords:** kefir yeast, *Kluyveromyces marxianus*, probiotics, survivability, gastrointestinal environment

## Abstract

*Kluyveromyces marxianus* accounts for > 90% of the yeast population of kefir, and recently, its probiotic potential has been actively explored with a focus on its health benefits and safety. Herein, the survivability of five kefir-isolated *K. marxianus* strains (Km A1–A5) in a simulated gastrointestinal (GI) environment was evaluated and compared with those of commercial probiotic yeast, *Saccharomyces boulardii* MYA-796. To further explore the potential to survive in the host GI tract, biochemical activities, hydrophobicity assay, biofilm formation, auto-aggregation analysis, and phenol tolerance of the strains were assessed. *K. marxianus* A4 exhibited the best survivability among all tested strains, including the clinically proven probiotic yeast strain *S. boulardii* MYA-796 (*p* = 0.014) in the artificial GI tract ranging from pH 2.0 to 7.5. In addition, the five *K. marxianus* strains and *S. boulardii* MYA-796 displayed different assimilation of lactose, xylitol, D-sorbitol, and DL-lactate, indicating that *K. marxianus* metabolized a wide range of substances and, thus, might be more feasible to nourish themselves in the host GI tract for survival. *K. marxianus* strains showed a greater hydrophobicity of cell surface, abilities to biofilm formation and auto-aggregation, and phenol tolerance than *S. boulardii* MYA-796, suggesting greater potential for survival in the host GI tract.

## Introduction

The term “probiotic” means “for life” and refers to live microorganisms providing health benefits to the host in adequate amounts (Quigley, 2019). The basic requirements for good probiotics include survivability in the host gastrointestinal (GI) tract, beneficial health effects, and non-pathogenicity (Kim et al., 2019b).

To date, lactic acid bacteria have been the most prominent and renowned probiotic microorganisms. However, recently, yeasts have received increasing attention as promising probiotics, mainly due to their better resistance to various environmental stresses, lower possibility of acquisition and transfer/distribution of antibiotic resistance, and differential immune signaling to the host when compared to lactic acid bacterial probiotics (Tambekar and Bhutada, 2010). In addition to these advantages, many studies over the past decades have explored the potential of novel yeast species as probiotic microorganisms (Kelesidis and Pothoulakis, 2012). For instance, *Saccharomyces boulardii* is widely accepted as a probiotic yeast that has been proven prevention of acute diarrhea in double-blind experiments (Sazawal et al., 2006; Czerucka et al., 2007; Organic Materials Review Institute [OMRI], 2014).

Kefir is a traditional dairy product containing multiple probiotic microorganisms, primarily lactic acid bacteria, such as *Lactobacillus kefiranofaciens* and *Lactobacillus kefiri*, and yeasts including *Kluyveromyces marxianus* and *Saccharomyces unisporus* (Kim et al., 2019a). Among these, *K. marxianus* has been reported as the major yeast population in kefir (Kim et al., 2015, 2020). Several studies have investigated the probiotic attributes of *K. marxianus*, including its adhesion to the intestinal epithelium, antagonism toward pathogenic bacteria, anti-microbial, and anti-inflammatory functions (Lane and Morrissey, 2010; Liu et al., 2012; Romanin et al., 2016). Our previous study also evaluated the safety of *K. marxianus* by characterizing its phenotypic traits associated with virulence, such as the formation of pseudo-hyphae, production of proteinases, and hemolytic activity (Lim et al., 2019; Youn et al., 2022).

Probiotic strains to be applied to hosts evaluate not only particular functional attributes but also typical attributes under extreme conditions such as survival and maintenance in the artificial GI tract and phenolic environments (Bao et al., 2010). There are various ways to simulate the survivability of potential probiotic agents in GI environment, including acid and bile tolerances, mimicking physical conditions of GI tract, and persistence during gut transit in animal models (Diosma et al., 2014; Cudennec et al., 2015; Kim et al., 2017). In addition, adhesion to intestinal cells may also reflect the same properties because probiotics can survive in the host. The hydrophobicity of a cell may depend on the expression of various surface-associated proteins between strains and change with variation in the physiological state of cells and strains of species (Nwanyanwu et al., 2012). A high percentage of biofilm formation and auto-aggregation of cells contribute to survival in the host GI tract and confer a competitive advantage over enteric bacterial pathogens (Suvarna et al., 2018). Furthermore, the auto-aggregation provides a protective barrier when probiotic strains form biofilms in the host GI tract and exopolysaccharides produced during biofilm formation inhibit pathogenic bacteria (Dertli et al., 2015). As these properties determined by the hydrophobicity of cell surface contribute to the activation of probiotics in humans, they are used to screen potential probiotics (Pan’kova et al., 2011). Relatively few studies have been conducted focusing on the survivability and the factors involved in the survival of the *K. marxianus* strains compared to the studies on the same aspects of *S. boulardii*.

In this study, we aimed to evaluate the survivability of five *K. marxianus* strains isolated from kefir in a simulated GI environment mimicking various physical conditions. Moreover, biochemical characteristics, hydrophobicity, biofilm formation, auto-aggregation, and phenol tolerance, that might affect their survivability in the GI environment, were also analyzed to further explore any correlations among them.

## Materials and Methods

### Experimental Design

**FIGURE 1:**
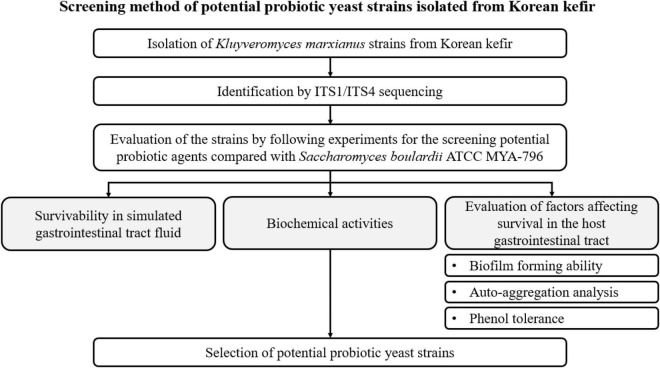
Flowchart of the experimental procedures in the present study.

A schematic flowchart of the experimental procedures used to screen potential probiotic yeast agents isolated from Korean kefir is shown in Figure 1.

### Isolation of Yeast Strains From Kefir

As a fermentation starter, kefir grain was obtained from the Center for One Health, Konkuk University, South Korea. Kefir was prepared by adding 50 g of viable kefir grains to 1 L of sterilized milk (Seoul Milk, Seoul Milk Cooperative, Seoul, South Korea) and fermenting the mixture at 25°C for 24 h. Next, the grains and milk were separated using a sterilized plastic filter (2 mm pore size). Kefir milk was prepared daily during the experimental period. To isolate *K. marxianus* strains from kefir, we streaked kefir milk on potato dextrose agar (PDA; Oxoid, Basingstoke, Hampshire, United Kingdom) and incubated aerobically at 30°C for 72 h. *S. boulardii* ATCC MYA-796 (Sb MYA-796) strain was kindly proved by prof. Dr. Hong-Gu Lee, College of Animal Bioscience & Technology, Konkuk University (Seoul, South Korea). Sb MYA-796 was used as a control strain since it is the most studied and only clinically demonstrated probiotic yeast strain (Sazawal et al., 2006). Strains were grown on PDA at 30°C for 24 h, and the cells were then sub-cultured twice under aerobic conditions.

### DNA Extraction

Genomic DNA was extracted using the NucliSENS easyMAG instrument (bioMérieux, Marcy l’Etoile, France) in accordance with the manufacturer’s instructions. Briefly, each colony was lysed in 1 mL of lysis buffer, and the lysate was incubated at 25°C for 30 min. The lysed sample was then transferred to a plastic vessel containing 50 μL of magnetic silica and subjected to automatic magnetic bead separation. The extracted DNA was resuspended in 25 μL of elution buffer.

### Identification of Yeast Strains

Five *K. marxianus* strains were identified via sequencing of the internal transcribed spacer (ITS) region (Diosma et al., 2014). Briefly, the primer pair sequences for ITS sequencing are as follows: ITS1: 5′- TCC GTA GGT GAA CCT GCG G-3′ and ITS4: 5′- TCC TCC GCT TAT TGA TAT GC-3′. Polymerase chain reaction (PCR) products were sequenced using the same primers and the ABI BigDye Terminator v3.1 Cycle Sequencing Kit (Applied Biosystems Carlsbad, CA, United States) according to the manufacturer’s instructions. Sequencing was performed with the initial denaturation at 94°C for 5 min, 35 cycles of denaturation at 94°C for 1 min, annealing at 56°C for 1 min, and extension at 72°C for 2 min followed by a final extension at 72°C for 10 min using an Applied Biosystems 3730XL DNA Analyzer by Bionics Co., Ltd (Seoul, South Korea). Obtained ITS sequences were aligned for each strain and were subjected to BLAST^1^ using the NCBI rRNA/ITS database for identification at the species level. The identities of the isolates were determined on the highest BLAST score.

### Evaluation of Survivability/Growth Potential in Simulated Gastrointestinal Tract Conditions

Survival of the yeast strains in the simulated GI tract was evaluated according to previous reports with some modifications (Cudennec et al., 2015; Ceugniez et al., 2017). The composition of the simulated fluids is given in Supplementary Table 1. Briefly, five *K. marxianus* and Sb MYA-796 were each suspended in potato dextrose broth (PDB; Difco, Detroit, MI, United States) and adjusted to 2.5 McFarland [approximately 10^5^ colony forming units (CFU)/mL]. The yeast strains prepared at 10^5^CFU/mL were added to the gastric fluid adjusted to pH 2.0 and 3.0, respectively. The gastric and intestinal fluids were incubated aerobically for 2 h or anaerobically for 24 h, respectively, with agitation at 80 rpm and 37°C. The gastric fluid containing yeasts were then diluted at a 1:1 ratio using the intestinal fluid adjusted to pH 7.5 and 8.5 (simulated serial GI environment I, pH 2.0 for gastric and 7.5 for the intestinal environment; simulated serial GI environment II, pH 2.0 for gastric and 8.5 for the intestinal environment; simulated serial GI environment III, pH 3.0 for gastric and 7.5 for the intestinal environment, and GI environment IV; pH 3.0 for gastric and 8.5 for the intestinal environment) to provide the test strains with sequential exposure to gastric and intestinal environments. The samples were serially diluted with sterile phosphate-buffered saline (PBS; Sigma-Aldrich, St. Louis, MO, United States) before (for enumeration of initially viable cells) and after treatment (for enumeration of final viable cells), and viable colonies were enumerated on PDA (Oxoid) for 24 h at 37°C.

### Biochemical Analysis of Yeast Strains

The biochemical analysis of the yeast species was conducted using the VITEK^®^ 2 system (bioMérieux) in accordance with the manufacturer’s instructions. Briefly, colonies of each yeast strain were suspended in 0.45% saline and allowed to approach McFarland 2.0 turbidity. Then, the suspension was placed in the VITEK^®^ 2 system and analyzed using the YST card (bioMérieux). YST cards were incubated at 35.5°C for 18 h, with optical readings automatically obtained every 15 min.

### Evaluation of Factors Affecting Survival in the Host Gastrointestinal Tract

#### Hydrophobicity Assay

The hydrophobicity of the five *K. marxianus* strains and Sb MYA-796 was evaluated using the microbial adhesion to solvent (MATS) method described by Bellon-Fontaine et al. (1996). The hydrophobic solvents used were hexadecane (analytical standard grade, 99.8%, Sigma-Aldrich, St. Louis, MO, United States), decane (anhydrous grade, 99%, Sigma-Aldrich), and chloroform (anhydrous grade, 99%, Sigma-Aldrich). In brief, the yeast strains were cultivated in PDB (Difco) at 37°C for 24 h and centrifuged at 7,280 × *g* for 10 min. The resulting pellet was washed twice with sterile PBS (Sigma-Aldrich) and resuspended in PBS at approximately 10^5^ CFU/mL. The optical density (OD) of the suspension was measured (A0) at a wavelength of 405 nm using a microplate reader (Multiskan FC, Thermo Fisher Scientific, Shanghai, China). Next, 280 μL of each yeast suspension was vortexed for 90 s with 40 μL of each solvent. The mixture was allowed to stand for 15 min to ensure complete separation of the two phases. The OD of the water phase was then measured (A1) at a wavelength of 405 nm. The percentage of cell surface hydrophobicity was calculated using the following equation:


Hydrophobicity(%)=(1-A1/A0)×100%

The percentage of hydrophobicity was expressed as follows: 0–35%, low hydrophobicity; 36–70%, medium hydrophobicity; and 71–100%, high hydrophobicity (Mladenović et al., 2020).

#### Biofilm Formation

The biofilm-forming ability of the yeast strains was evaluated as previously described (Jeong et al., 2018). In brief, each colony of the isolates was added to 200 μL PDB (Difco) and set to 3.0 McFarland turbidity. To evaluate the biofilm formation, 200 μL of each sample was transferred to a 96-well polystyrene culture plate (SPL Life Sciences, Gyeonggi-do, South Korea) and incubated at 37°C for 24 h. The culture medium was discarded, and the microplate was gently washed twice with 200 μL PBS (Sigma-Aldrich). Cells were stained with 0.1% (w/v) crystal violet (100 μL; Sigma-Aldrich, St. Louis, MO, United States) for 15 min at room temperature (20–25°C) and rinsed twice with PBS (Sigma-Aldrich). After removing the dye with 200 μL of 99% ethanol, the biofilm was quantified by measuring the absorbance at 595 nm using a Multiskan FC (Thermo Fisher Scientific).

#### Auto-Aggregation Assay

Auto-aggregation assay was performed according to the method of Fonseca et al. (2021) with slight modifications. Briefly, yeast strains were pelleted in PBS (Sigma-Aldrich) and adjusted to obtain 10^5^ CFU/mL in the same buffer. The OD of the suspension before (A0) and after 5 h incubation at 37°C (At) were measured at a wavelength of 595 nm using a Multiskan FC (Thermo Fisher Scientific). The incubation time of At was set in consideration of the lag phases of *K. marxianus* and *S. boulardii* to prevent the mistaken result of auto-aggregation due to the growth of the yeast strains. The plate was shaken for 5 sec immediately before each reading. The auto-aggregation percentage was determined using the following equation:


Auto-aggregation(%)=(1-At/A0)×100%

#### Phenol Tolerance

Phenol tolerance of yeast strains was evaluated as described by Shehata et al. (2016) with slight modifications. Overnight cultures of yeast strains were inoculated (1%) in PDB (Difco) with 0.2 and 0.5% v/v of phenol or without phenol. Yeast cells in the PDB were quantified by reading the OD_620_ after 24 h incubation at 37°C.

### Statistical Analyses

**FIGURE 2:**
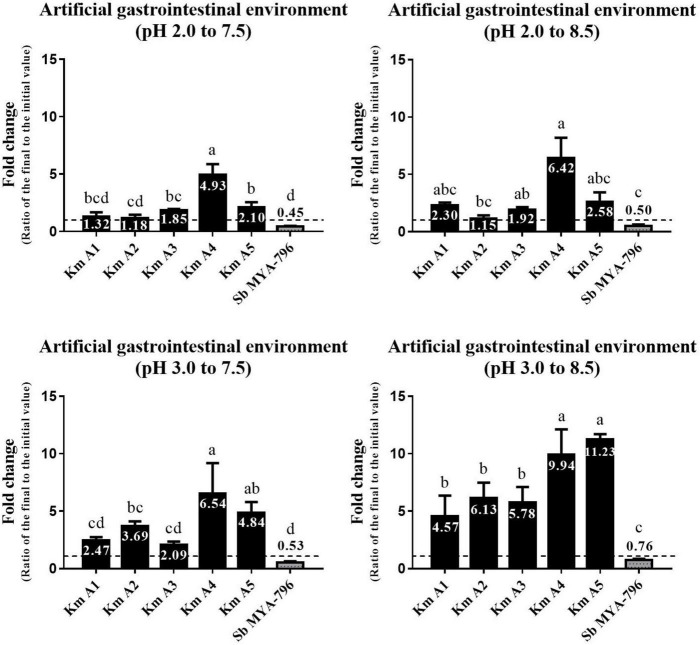
Changes in the number of viable cells of the five *Kluyveromyces marxianus* strains isolated from kefir and *Saccharomyces boulardii* MYA-796 at pH 2.0 to 7.5, 2.0 to 8.5, 3.0 to 7.5, and 3.0 to 8.5. Different letters above the bars indicate significant differences at *p* < 0.05 using the Duncan method. The yeast strains were exposed to the simulated gastric fluid adjusted to pH 2.0 and 3.0 and incubated with agitation for 2 h at 37°C, respectively. Then, the yeast-containing gastric fluids were diluted using intestinal fluids adjusted to pH 7.5 and 8.5 to provide sequential exposure to gastric and intestinal environments and incubated with agitation for 24 h at 37°C, respectively. All experiments were performed in triplicate. **Km A1**, *K. marxianus* A1; **Km A2**, *K. marxianus* A2; **Km A3**, *K. marxianus* A3; **Km A4**, *K. marxianus* A4; **Km A5**, *K. marxianus* A5; **Sb MYA-796**, *S. boulardii* MYA-796.

**FIGURE 3:**
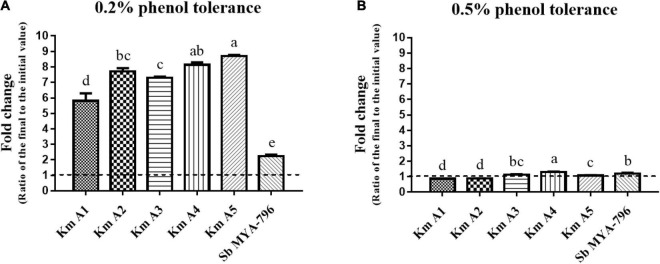
Effect of phenol concentration on the growth of the *Kluyveromyces marxianus* strains and *Saccharomyces boulardii* MYA-796. **(A)** The 0.2% phenol tolerance and **(B)** 0.5% phenol tolerance of all yeast strains. Different letters above the bars at the same phenol tolerance indicate significant differences at *p* < 0.05 using the Duncan method. All experiments were performed in triplicate. **Km A1**, *K. marxianus* A1; **Km A2**, *K. marxianus* A2; **Km A3**, *K. marxianus* A3; **Km A4**, *K. marxianus* A4; **Km A5**, *K. marxianus* A5; **Sb MYA-796**, *S. boulardii* MYA-796.

All experiments were performed in triplicate. SPSS version 25.0 (SPSS Inc., Chicago, IL) was used for data analysis. The final yeast cell count was divided by the initial cell count to express the survivability/growth potential and phenol tolerance (fold change); in Figures 2, 3, values below 1.0 indicated survivability, and those over 1.0 indicated fold-growth. All data were analyzed for normal distribution, and homogeneity of variance was conducted using one-way analysis of variance (ANOVA), followed by Duncan’s *post hoc* analysis. Differences were considered significant at *p* < 0.05.

## Results

### Yeast Strain Identification

Intezrnal transcribed spacer (ITS) sequencing identified *K. marxianus* at the species level and their sequences (Accession number of MT791345 for *K. marxianus* A1; MT793595 for *K. marxianus* A2; MT793593 for *K. marxianus* A3; MT793596 for *K. marxianus* A4; and MT793594 for *K. marxianus* A5) were submitted to GenBank.

### Survival in Artificial Gastrointestinal Tract Fluid

Five *K. marxianus* strains (Km A1-A5) and Sb MYA-796 were evaluated for their survivability in the simulated GI environment. *K. marxianus* strains grew in number ranging from 1.15 to 11.23-fold in various GI environments (Figure 2). In general, Km A4 and A5 showed the highest survivability among Km strains tested. In contrast, Sb MYA-796 did not show growth but only survived, ranging from 0.45 to 0.76-fold in various environments. Interestingly, Km A4 showed significantly higher survivability than Sb MYA-796 in all simulated serial GI environments (*p* < 0.05). All yeast strains showed higher survivability or fold-growth in the environment with higher pH scales (i.e., from the lowest pH scale of GI environment I to the highest of GI environment IV).

### Biochemical Activities

Supplementary Table 2 represents the full list of the 46 biochemical characteristics of Km A1-A5 isolated from kefir as well as those of Sb MYA-796, evaluated using the VITEK^®^ 2 System. Overall, compared to Sb MYA-796, all *K. marxianus* strains displayed a broader range of biochemical activities: the number of positive/total tests (%) were 16/46 (34.78%), 17/46 (36.96%), 14/46 (30.43%), 16/46 (34.78%), 18/46 (39.13%), and 11/46 (23.91%) for Km A1-A5 and Sb MYA-796, respectively. Selected biochemical tests are represented in Table 1. The biochemical activities present in all *K. marxianus* strains and absent in Sb MYA-796 included lactose assimilation, xylitol assimilation, D-sorbitol assimilation, and DL-lactate assimilation (Table 1). Conversely, biochemical activities absent in all *K. marxianus* strains and present in Sb MYA-796 included D-maltose assimilation, D-turanose assimilation, and D-trehalose assimilation (Table 1).

**TABLE 1:** Selected biochemical activities of the five *Kluyveromyces marxianus* strains isolated from kefir and *Saccharomyces boulardii* MYA-796 differing at the species or strain levels, as analyzed using the VITEK^®^ 2 system.

Associated metabolism	Biochemical test	Yeast strain					
		Km A1	Km A2	Km A3	Km A4	Km A5	Sb MYA-796
Carbohydrate metabolism	Lactose assimilation (LACa)^†^	+	+	+	+	+	–
	Xylitol assimilation (XLTa)^†^	+	+	+	+	+	–
	D-sorbitol assimilation (dSORa)^†^	+	+	+	+	+	–
	DL-lactate assimilation (LATa)^†^	+	+	+	+	+	–
	D-maltose assimilation (dMALa)^†^	–	–	–	–	–	+
	D-turanose assimilation (dTURa)^†^	–	–	–	–	–	+
	D-trehalose assimilation (dTREa)^†^	–	–	–	–	–	+
	L-malate assimilation (IMLTa)^‡^	+	+	–	–	+	–
	Amygdaline assimilation (AMYa)^‡^	+	+	–	+	+	+
	L-arabinose assimilation (IARAa)^‡^	–	–	+	+	+	–
	D-xylose assimilation (dXYLa)^‡^	–	+	+	+	+	–
Protein metabolism	Urease (URE)^‡^	–	–	–	+	–	–
	L-glutamate assimilation (IGLTa)^‡^	+	+	+	–	+	–
	L-proline assimilation (IPROa)^‡^	+	+	–	–	+	–
	Tyrosine-arylamidase (TyrA)^‡^	+	+	–	+	+	–

*^†^Biochemical activities of five K. marxianus strains and S. boulardii MYA-796 differing at the species level.*

*^‡^Biochemical activities of five K. marxianus strains and S. boulardii MYA-796 differing at the strain level.*

***Km A1**, K. marxianus A1; **Km A2**, K. marxianus A2; **Km A3**, K. marxianus A3; **Km A4**, K. marxianus A4; **Km A5**, K. marxianus A5; **Sb MYA-796**, S. boulardii MYA-796.*

Differing biochemical activities at the strain level were as follows: L-malate assimilation, amygdaline assimilation, L-arabinose assimilation, D-xylose assimilation, urease being positive only in Km A4, L-glutamate assimilation, L-proline assimilation, and tyrosine-arylamidase (Table 1).

### Evaluation of Factors Affecting Survival in the Host Gastrointestinal Tract

#### Hydrophobicity Analysis

The hydrophobicity of the cell surface of tested yeasts was evaluated using three hydrophobic solvents, including hexadecane, decane, and chloroform (Table 2). All *K. marxianus* strains had higher adhesion to hydrophobic solvents than Sb MYA-796, which exhibited medium hydrophobicity to all solvents. On the other hand, Km A2, A4, and A5 displayed high hydrophobicity with more than 70% adhesion. Interestingly, the most hydrophobic strain was Km A4, showing a significantly high affinity to hexadecane, decane, and chloroform (*p* < 0.05).

**TABLE 2:** Adhesion properties of *Kluyveromyces marxianus* strains isolated from kefir compared with that of *Saccharomyces boulardii* MYA-796.

Yeast strains	Hydrophobicity (%)		
	Hexadecane	Decane	Chloroform
Km A1	66.95 ± 3.41^c^	71.41 ± 1.37^b^	69.05 ± 0.88^d^
Km A2	72.04 ± 1.24^b^	71.67 ± 0.62^b^	73.91 ± 4.35^c^
Km A3	67.89 ± 2.71^c^	60.93 ± 1.11^c^	65.38 ± 3.55^e^
Km A4	76.89 ± 4.03^a^	73.80 ± 1.21^a^	82.18 ± 3.55^a^
Km A5	75.02 ± 2.14^ab^	70.11 ± 2.07^b^	77.88 ± 2.50^b^
Sb MYA-796	59.44 ± 1.81^d^	60.28 ± 0.67^c^	59.36 ± 1.99^f^

*Different letters in a column indicate significant differences (p < 0.05) using the Duncan method. Km A1, K. marxianus A1; Km A2, K. marxianus A2; Km A3, K. marxianus A3; Km A4, K. marxianus A4; Km A5, K. marxianus A5; Sb MYA-796, S. boulardii MYA-796.*

#### Biofilm-Forming Ability

**FIGURE 4:**
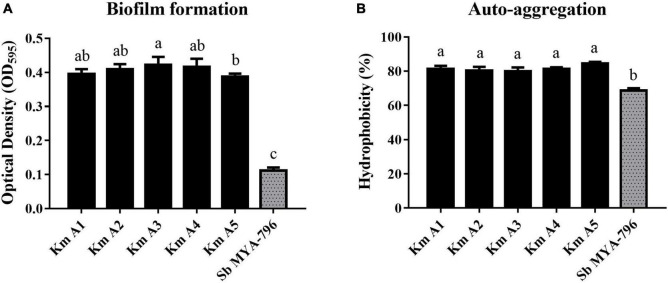
**(A)** Biofilm formation and **(B)** auto-aggregation analysis of *Kluyveromyces marxianus* strains (Km A1–A5) compared with those of *Saccharomyces boulardii* MYA-796 at 37°C. Error bars represent standard deviations. Different letters indicate statistical differences at *p* < 0.05 using the Duncan method. All experiments were performed in triplicate. **NC**, Negative control (Potato dextrose broth); **Km A1**, *K. marxianus* A1; **Km A2**, *K. marxianus* A2; **Km A3**, *K. marxianus* A3; **Km A4**, *K. marxianus* A4; **Km A5**, *K. marxianus* A5; **Sb MYA-796**, *S. boulardii* MYA-796.

The biofilm-forming ability of Km A1-A5 was significantly different from that of Sb MYA-796 after 24 h at 37°C (*p* < 0.05; Figure 4A). The mean OD_595_ value of Km A1-A5 was observed to be 0.3960, 0.4098, 0.4226, 0.4160, and 0.3874, respectively, whereas Sb MYA-796 showed a weaker capacity to form biofilms (mean OD_595_ value of 0.1118) than all *K. marxianus* strains.

#### Auto-Aggregation Analysis

The yeast strains evaluated in the present study had values of auto-aggregation ranging from 68.67 to 84.66% after 5 h of incubation (Figure 4B). The highest values were found for Km A5, which exhibited an auto-aggregation value of 84.66%, whereas that of Sb MYA-796 was 68.67% (*p* < 0.05).

#### Phenol Tolerance

The effect of different phenol concentrations (0.2 and 0.5%) on the growth of Km A1-A5 and Sb MYA-796 was determined, as shown in Figure 3. All strains were more tolerant to 0.2% phenol than 0.5% phenol. In 0.2% phenol solution, Km A5 (8.70-fold) was the most tolerant, followed by Km A4 (8.14-fold), whereas Sb MYA-796 (2.24-fold) had the lowest value compared to that of Km A1-A5 (*p* < 0.05, Figure 3A). In 0.5% phenol solution, Km A4 was the most tolerant, exhibiting 1.07-fold survivability compared to the other yeast strains (*p* < 0.05, Figure 3B).

## Discussion

To the best of our knowledge, few studies have demonstrated the survivability and survival affecting factors of *K. marxianus* isolated from kefir *in vitro* with the most renowned probiotic yeast, *S. boulardii*, as a reference. The results highlighted greater survivability/growth potential of *K. marxianus* in the simulated serial GI environments and a broader range of biochemical activities compared to Sb MYA-796. In addition, the hydrophobicity of cell surface, auto-aggregation, biofilm formation, and phenol tolerance of the *K. marxianus* strains were superior to these of *S. boulardii* MYA-796.

A previous study reported that the survivability of *K. marxianus* isolated from kefir in artificial gastric and intestinal environments for 30 min and 6 h, respectively, was superior to that of *Saccharomyces cerevisiae* KCTC 7004 (You et al., 2006). Moreover, *K. marxianus* S97, S101, and S106 isolated from fruits and dairy samples survived approximately log 6.8 CFU/mL after exposure to pH 2.0 for 96 h and showed higher survivability than *S. cerevisiae* S28 and S34 (approximately log 6.0 CFU/mL) (Moradi et al., 2018). Another study suggested that, under simulated gastric conditions (pH 3.0) and duodenum juice, *K. marxianus* NS1KM2, 14KM1, and 6688 KM isolated from Fiore Sardo cheese had better survival (decrease by 0–17%) than *S. boulardii* CODEX SB1 (decrease by 64.5%) (Fadda et al., 2017). Especially, in a recent study (Ceugniez et al., 2017), the survivability of *K. marxianus* S-2-05 decreased by 0.47-fold in a consecutive simulated serial GI environment (pH 3.0 to 7.0; 2 h for gastric condition and 2 h for intestinal condition), whereas in this study, Km A4 and Km A5 increased by 6.54 and 4.84-fold, respectively, in the same pH environment (pH 3.0 to 7.0; 2 h for gastric condition and 24 h for intestinal condition). The different survivability of yeast strains in the two studies is due to the different incubation times of simulated intestinal environments. Comparing viability for more than 12 h in an intestinal environment is important because it shows that microorganisms can not only survive but also grow in the environment. However, no previous studies have focused on the survivability or growth potential of *K. marxianus* versus *S. boulardii* over a wide range of pH in a consecutive simulated GI environment (2 h for gastric condition and 24 h for intestinal condition). Here, we demonstrated that both *K. marxianus* and *S. boulardii* survived in the mimicking host GI tract; however, *K. marxianus* strains were more resistant than those of *S. boulardii*. The excellent survivability of *K. marxianus* evaluated in this study could benefit industries looking for yeast probiotics that can survive against preservatives other than Sb MYA-796.

Among kefir ecosystems, kefir yeasts appear to exert superior survivability to kefir lactic acid bacteria. Numerous survivability studies conducted on probiotic lactic acid bacteria have shown the relatively low survivability of these microorganisms; for example, *Lactobacillus acidophilus* M23 displayed a reduction of 4.1 log CFU/mL in a gastric environment at pH 2.5 (Song et al., 2015). In contrast, all *K. marxianus* strains displayed a reduction of less than 1 log CFU/mL under highly acidic conditions. We previously reported that *L. kefiranofaciens*, *Leuconostoc mesenteroides*, and *L. kefiri* strains, isolated from kefir, not only survived but also grew to exceed the initial bacterial count in the gastric environment adjusted to pH 2.5 with pepsin and intestinal environment adjusted to pH 7.0 with 0.3% oxgall, respectively (Kim et al., 2017). However, *Lactococcus lactis* strains displayed < 50% survival in the gastric environment (pH 2.5, 1000 U/mL pepsin for 2 h) as well as in the intestinal environment (pH 8.0, 0.3% oxgall for 24 h) (Kim et al., 2017). In comparison with these historical data, the superior survivability of *K. marxianus* strains might partially support our recent findings that Km A5 exerted better competitive exclusion against *Salmonella enterica* serovar Enteritidis than *L. kefiranofaciens* DN1 in the GI tract of chicks (Bae et al., 2020).

Several studies have investigated the biochemical and metabolic aspects of different *K. marxianus* strains for potential application in a bioindustrial reactor rather than as probiotics (Fonseca et al., 2008). To date, many studies have explored the potential benefits of probiotic attributes of *Lactobacillus* and *Bifidobacterium* spp. on the host digestive system, such as enzymatic capacity, modulation of the metabolic functioning of the host, and alleviating the symptoms of several diseases (Rabot et al., 2010; Sánchez et al., 2017) using animal models and clinical interventions, neglecting the importance of the biochemical activities as a survival factor of the probiotic microorganism itself. Microorganisms that use a wide range of metabolic substrates are more likely to survive by decomposing the complex substrates into intermediate fermentation products, including fumarate, succinate, and lactate in the host GI tract and using them to obtain energy (Rowland et al., 2018). In this light, *K. marxianus* with a wide spectrum of biochemical activities might be helpful for the survivability of the host GI tract (Rabot et al., 2010).

*K. marxianus* constitutes the majority of lactose-utilizing yeasts in dairy products such as milk, and all *K. marxianus* strains tested were positive for lactose assimilation (Simova et al., 2002; Diosma et al., 2014). This ability could be attributed to two genes, *lac*4 and *lac*12, which encode a β-galactosidase and lactose permease, respectively (Lane and Morrissey, 2010). β-galactosidase from *Kluyveromyces* spp. and the filamentous fungus *Aspergillus niger* is the most common form of commercial lactase (Adam et al., 2005). Therefore, *K. marxianus* strains could alleviate lactose intolerance and more survive in the host GI tract than *Saccharomyces* spp. In different biochemical activities at the strain level, urease was positive only in Km A4, which showed the highest survivability in the most acidic gastric environment. Urease is considered a stress response that counteracts the effects of low pH environments and modulates intracellular and extracellular pH in some bacteria (Mora et al., 2004). Colonization of *Helicobacter pylori* in the stomach has been related to the presence of bacterial urease and urea metabolism (Ferrero et al., 1988). Probiotics such as *Bifidobacterium longum* subsp. *infantis* and *Streptococcus thermophilus* are urease-positive bacteria that colonize early in the GI tract of the host (Mora et al., 2004; LoCascio et al., 2010). Moreover, these urease-positive microorganisms share the environmental benefit of a temporal local pH increase with urease-negative microorganisms (Arioli et al., 2010). The urease-positive Km A4 consistently presented the highest survivability in the most acidic gastric environment.

Hydrophobic cell surface was demonstrated by high adherence to hexadecane, decane, and chloroform. Many studies have shown that the presence of glycoprotein on the cell surface results in higher hydrophobicity (Cuperus et al., 1993). In a previous study, when the hydrophobic ability was set as an affinity higher than 40% in hexadecane and chloroform, *Lactobacillus paracasei* lac 1, *L. acidophilus* lac 2 and 3, and *Lactobacillus plantarum* lac 6 showed hydrophobicity of the cell surface (Abdulla et al., 2014). According to another study, the affinity of *L. acidophilus* M92 and *L. plantarum* L4 for chloroform was 36.06% and 47.03%, respectively (Kos et al., 2003). After comparison with a previous study, the biofilm formation ability of *K. marxianus* strains (Km A1-A5) isolated from kefir was superior to that of *K. marxianus* S-2-05 isolated from a traditional French cheese. Moreover, *K. marxianus* S-2-05 had a low affinity for hexadecane (8.89%), decane (20.13%), and chloroform (60.41%) compared to the Km A1-A5, implying that the cell surface hydrophobicity and biofilm-forming ability depend on strain (Ceugniez et al., 2017). The relatively higher affinities to chloroform of kefir yeast strains indicate the basic character of the yeast cell, which is related to the presence of carboxylic groups on the microbial surface (Bellon-Fontaine et al., 1996). Our results indicate that *K. marxianus* strains isolated from kefir have greater hydrophobicity, biofilm formation, and auto-aggregation properties than the probiotic Sb MYA-796.

Phenols are formed by bacterial degradation of the aromatic amino acids, inhibit bacteria in gut microbiota, and affect diversity and metabolic activity (Fonseca et al., 2021). Intestinal bacteria involved in phenolic formation include *Bacteroides*, *Enterobacteriaceae*, *Lactobacillus*, and *Bifidobacterium* (Hughes et al., 2000). In the gut environment, phenolic compounds may selectively inhibit or stimulate the growth of some of the intestinal microorganisms and can also affect bacteria population kinetics (Tzounis et al., 2008). Moreover, phenols have bacteriostatic effects against potential probiotic agents (Fonseca et al., 2021). Consistent results reported that a 0.4% phenol concentration causes a bacteriostatic action in *L. acidophilus* DC 602 and *L. gasseri* DC 422 (Xanthopoulos et al., 2000). Consequently, phenol tolerance is essential for the characterization of probiotic strains (Divisekera et al., 2019). To the best of our knowledge, this is the first study to demonstrate the phenol tolerance of potential probiotic yeast. Km A1–A5 showed a difference in sensitivity for different phenol concentrations but overall could tolerate the tested phenol concentrations. This could be noted given a recent report that three *Lactobacillus* spp. could not withstand 0.5% phenol (Divisekera et al., 2019).

A major limitation of this study is that we did not simulate the biological nor the physical conditions such as microbiome and lack of oxygen. Additionally, most factors affecting survival were evaluated under the standard conditions and not in the simulated GI environment; furthermore, visual and microscopic analyses were not conducted. Thus, further studies should aim to evaluate the survivability of the *K. marxianus* strains *in vivo* and microscopical analysis is needed to support the findings described in the present study.

## Conclusion

The survivability/growth potential of *K. marxianus* strains was greater than that of Sb MYA-796 under simulated GI conditions. This could be attributed to the extensive spectrum of biochemical activities of *K. marxianus* strains. It was also assumed that higher hydrophobicity, biofilm-forming and auto-aggregation abilities, as well as phenol tolerance in *K. marxianus* strains than Sb MYA-796, could strongly correlate with the superior survivability of *K. marxianus*. In conclusion, our study will provide a basis for understanding the correlations among the survivability and other characteristics the newly isolated *K. marxianus* strains from kefir.

## Data Availability Statement

The original contributions presented in the study are included in the article/Supplementary Material, further inquiries can be directed to the corresponding author.

## Author Contributions

H-YY: conceptualization, methodology, investigation, formal analysis, data curation, and writing—original draft. D-HK: conceptualization, methodology, data curation, isolation of *Kluyveromyces marxianus* strains, and writing—original draft. H-JK: investigation, formal analysis, and writing—original draft. DB and K-YS: writing—review and editing. HK: supervision, funding acquistion, and writing—review and editing. K-HS: supervision, funding acquisition, writing—review and editing, and project administration. All authors contributed to the article and approved the submitted version.

## Conflict of Interest

The authors declare that the research was conducted in the absence of any commercial or financial relationships that could be construed as a potential conflict of interest.

## Publisher’s Note

All claims expressed in this article are solely those of the authors and do not necessarily represent those of their affiliated organizations, or those of the publisher, the editors and the reviewers. Any product that may be evaluated in this article, or claim that may be made by its manufacturer, is not guaranteed or endorsed by the publisher.

## Footnotes

1
https://blast.ncbi.nlm.nih.gov/Blast.cgi
