# Supplementary material for: Survivability of Kluyveromyces marxianus Isolated From Korean Kefir in a Simulated Gastrointestinal Environment
Source: Front Microbiol. 2022 Feb 24;13:842097. doi: 10.3389/fmicb.2022.842097 (PMC8908258; doi:10.3389/fmicb.2022.842097)
Supplement: Supplementary file 1 [file Table_1.DOCX]

***Supplementary Material***

**1 Supplementary Tables**

**Supplementary Table 1**. Composition of the artificial gastrointestinal fluids.

| Compound | Gastric fluid | Intestinal fluid | Reference |
| --- | --- | --- | --- |
| KCl | 6.9 mM | 6.8 mM | Cudennec et al., 2015;  Ceugniez et al., 2017 |
| KH_2_PO_4_ | 0.9 mM | 0.8 mM |  |
| NaHCO_3_ | 24.5 mM | 85 mM |  |
| NaCl | 47.7 mM | 38.4 mM |  |
| MgCl_2_(H_2_O)_6_ | 0.105 mM | 0.33 mM |  |
| NH_4_HCO_3_ | 1 mM | - |  |
| HCl | 15 mM | 8.4 mM |  |
| CaCl_2_ | 0.15 mM | 0.6 mM |  |
| Pepsin (porcine) | 4000 U/mL | - |  |
| Pancreatin (porcine) | - | 100 U/mL |  |
| Bile extract (porcine) | - | 60 g/L |  |
| pH | 2.0 and 3.0 | 7.5 and 8.5 |  |

**Supplementary Table 2.** Biochemical activities of the five *K. marxianus* strains isolated from kefir and *Saccharomyces boulardii* MYA-796 analyzed using the VITEK^®^ 2 system

| **Biochemical test** | **Yeast strain** | | | | | |
| --- | --- | --- | --- | --- | --- | --- |
|  | **Km A1** | **Km A2** | **Km A3** | **Km A4** | **Km A5** | **Sb MYA-796** |
| L-lysine-arylamidase (LysA) | - | - | - | - | - | - |
| L-malate assimilation (IMLTa)^‡^ | + | + | - | - | + | - |
| Leucine-arylamidase (LeuA) | + | + | + | + | + | + |
| Arginine (ARG) | - | - | - | - | - | - |
| Erythritol assimilation (ERYa) | - | - | - | - | - | - |
| Glycerol assimilation (GLYa) | - | - | - | - | - | - |
| Tyrosine-arylamidase (TyrA)^‡^ | + | + | - | + | + | - |
| β-N-acetyl-glucosaminidase (BNAG) | - | - | - | - | - | - |
| Arbutin assimilation (ARBa) | - | - | - | - | - | - |
| Amygdaline assimilation (AMYa)^‡^ | + | + | - | + | + | + |
| D-galactose assimilation (dGALa) | + | + | + | + | + | + |
| Gentiobiose assimilation (GENa) | - | - | - | - | - | - |
| D-glucose assimilation (dGLUa) | + | + | + | + | + | + |
| Lactose assimilation (LACa)^†^ | + | + | + | + | + | - |
| Methyl-A-D-glucopyranoside (MAdGa) | - | - | - | - | - | - |
| D-cellobiose assimilation (dCELa) | - | - | - | - | - | - |
| Gamma-glutamyl-transferase (GGT) | - | - | - | - | - | - |
| D-maltose assimilation (dMALa)^†^ | - | - | - | - | - | + |
| D-raffinose assimilation (dRAFa) | + | + | + | + | + | + |
| PNP-N-acetyl-BD-galactosaminidase 1 (NAGA1) | - | - | - | - | - | - |
| D-mannose assimilation (dMNEa) | + | + | + | + | + | + |
| D-melibiose assimilation (dMELa) | - | - | - | - | - | - |
| D-melezitose assimilation (dMLZa) | - | - | - | - | - | - |
| L-sorbose assimilation (ISBEa) | - | - | - | - | - | - |
| L-rhamnose assimilation (IRHAa) | - | - | - | - | - | - |
| Xylitol assimilation (XLTa)^†^ | + | + | + | + | + | - |
| D-sorbitol assimilation (dSORa)^†^ | + | + | + | + | + | - |
| Sucrose assimilation (SACa) | + | + | + | + | + | + |
| Urease (URE)^‡^ | - | - | - | + | - | - |
| Alpha-glucosidase (AGLU) | - | - | - | - | - | - |
| D-turanose assimilation (dTURa)^†^ | - | - | - | - | - | + |
| D-trehalose assimilation (dTREa)^†^ | - | - | - | - | - | + |
| Nitrate assimilation (NO3a) | - | - | - | - | - | - |
| L-arabinose assimilation (IARAa)^‡^ | - | - | + | + | + | - |
| D-galacturonate assimilation (dGATa) | - | - | - | - | - | - |
| Esculin assimilation (ESC) | - | - | - | - | - | - |
| L- glutamate assimilation (IGLTa)^‡^ | + | + | + | - | + | - |
| D-xylose assimilation (dXYLa)^‡^ | - | + | + | + | + | - |
| DL-lactate assimilation (LATa)^†^ | + | + | + | + | + | - |
| Acetate assimilation (ACEa) | + | + | + | + | + | + |
| Citrate assimilation (CITa) | - | - | - | - | - | - |
| Glucuronate assimilation (GRTas) | - | - | - | - | - | - |
| L-proline assimilation (IPROa)^‡^ | + | + | - | - | + | - |
| 2-keto-D-gluconate assimilation (2KGa) | - | - | - | - | - | - |
| N-acetyl-glucosamine (NAGa) | - | - | - | - | - | - |
| D-gluconate assimilation (dGNTa) | - | - | - | - | - | - |
| Number of positive tests/Number of total tests (%) | 16/46 (34.78) | 17/46 (36.96) | 14/46 (30.43) | 16/46 (34.78) | 18/46 (39.13) | 11/46 (23.91) |

^†^Biochemical activities differing at the species level.

^‡^Biochemical activities differing at the strain level.

**Km A1**, *K. marxianus* A1; **Km A2**, *K. marxianus* A2; **Km A3**, *K. marxianus* A3; **Km A4**, *K. marxianus* A4; **Km A5**, *K. marxianus* A5; **Sb MYA-796**, *S. boulardii* MYA-796
